# Supplementary material for: Pistacia vera Extract Potentiates the Effect of Melatonin on Human Melatonin MT1 and MT2 Receptors with Functional Selectivity
Source: Pharmaceutics. 2023 Jun 28;15(7):1845. doi: 10.3390/pharmaceutics15071845 (PMC10386454; doi:10.3390/pharmaceutics15071845)
Supplement: Supplementary file 1 [file pharmaceutics-15-01845-s001.zip › pharmaceutics-2455253-supplementary.pdf]

Supplementary Figure 1

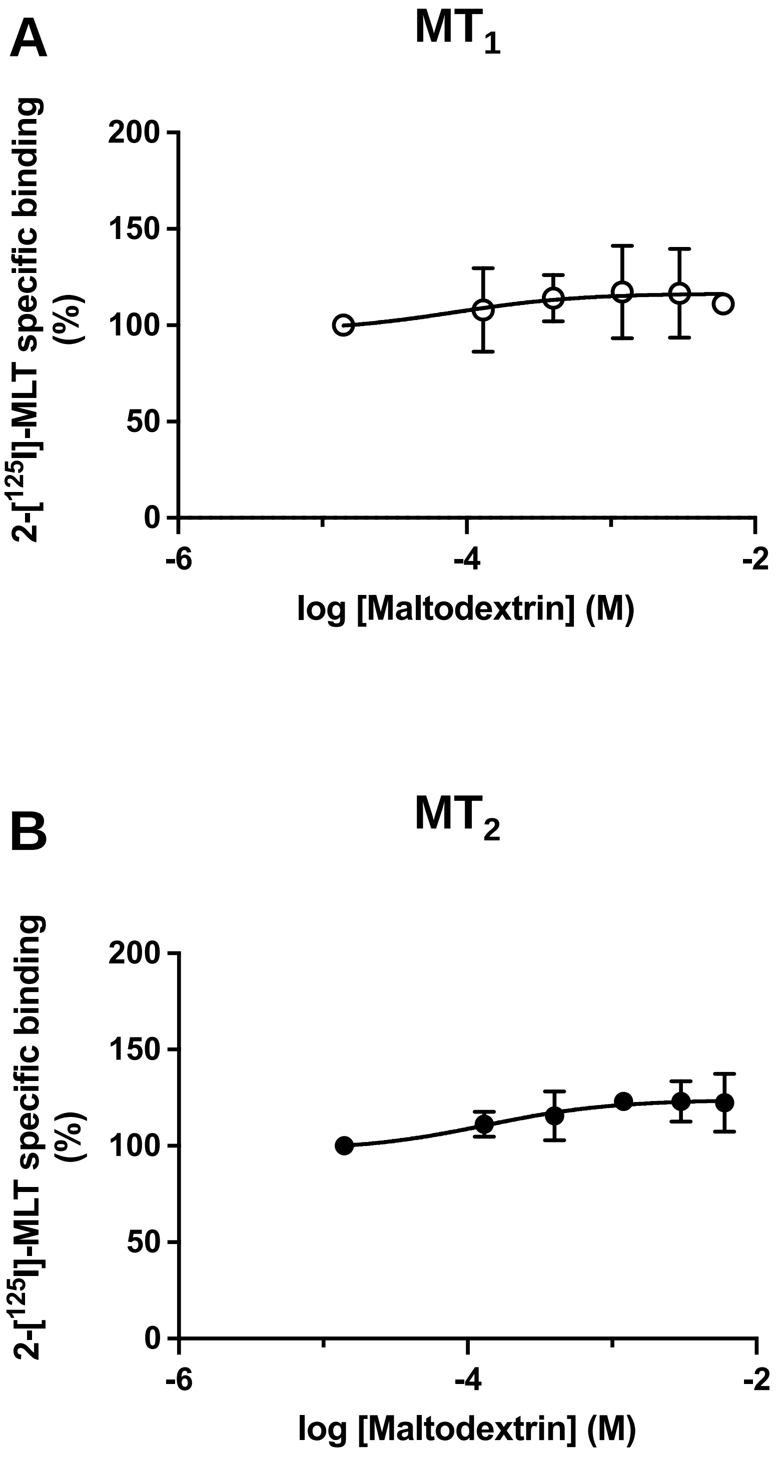

Supp Figure 1: **Effect of maltodextrin on MT_1 (A) and MT_2 (B) receptors**. Crude membrane preparations were incubated with increasing concentrations of maltodextrin and 100 pM of 2-[^{125}I]-MLT. Data are expressed as mean ± SEM, n = 2.

Supplementary Figure 2

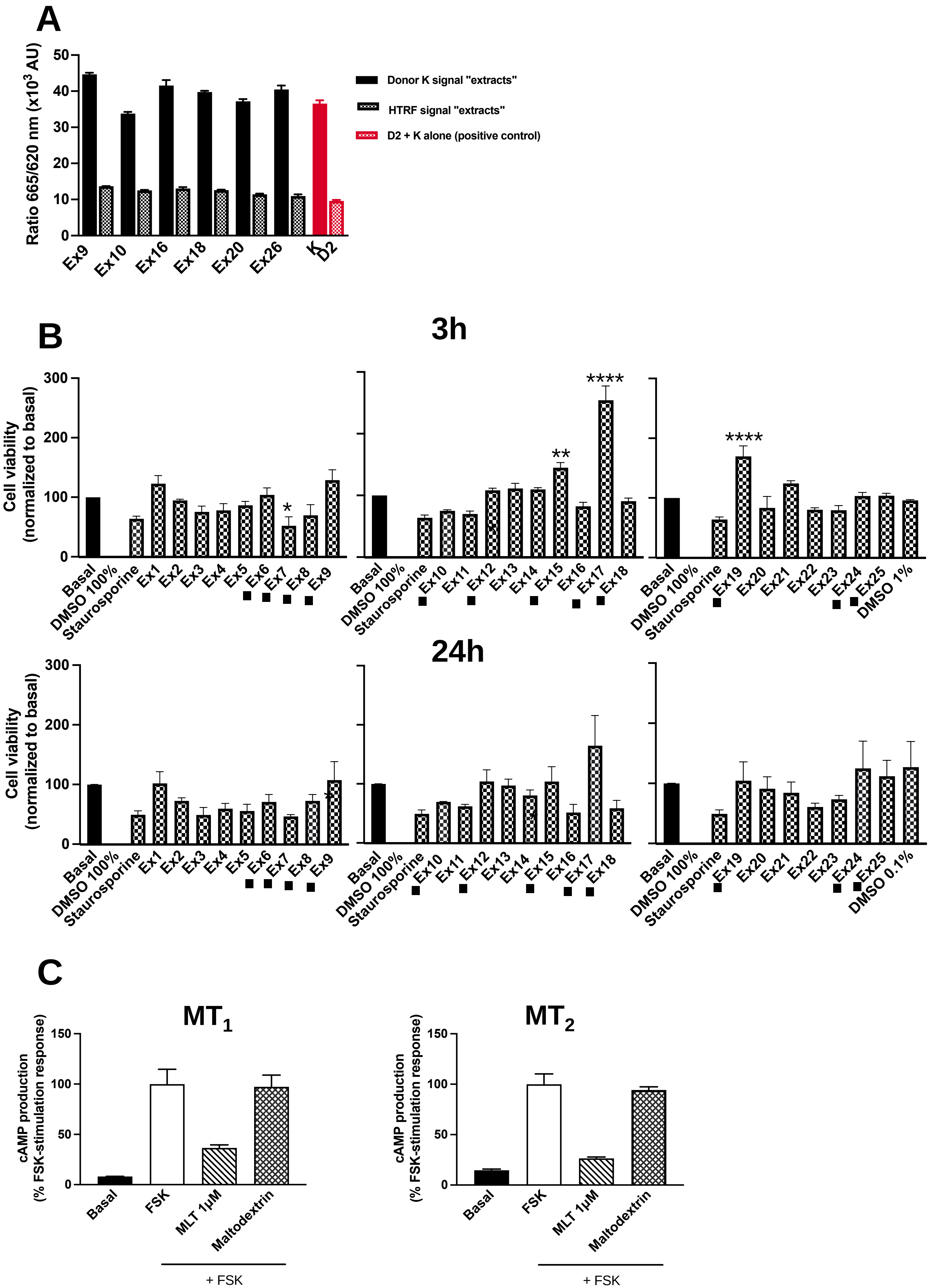

Supp. Figure 2. **Experimental controls of extracts.** The interference of extract colour (**A**), cell viability (**B**) and the maltodextrin additive (**C**) were evaluated in cAMP and MTT assays. All extracts and maltodextrin were tested at 0.16 mg/ml. **Ex18** was used at 0.005 mg/ml. Data are expressed as mean ± SEM of one experiment performed in triplicates (A,C) or 3 independent experiments performed in duplicates (B). (*p < 0.05; **p < 0.01; ****P <0.0001; One-way ANOVA). Extracts selected for functional studies are labelled with a black square.

Supplementary Figure 3

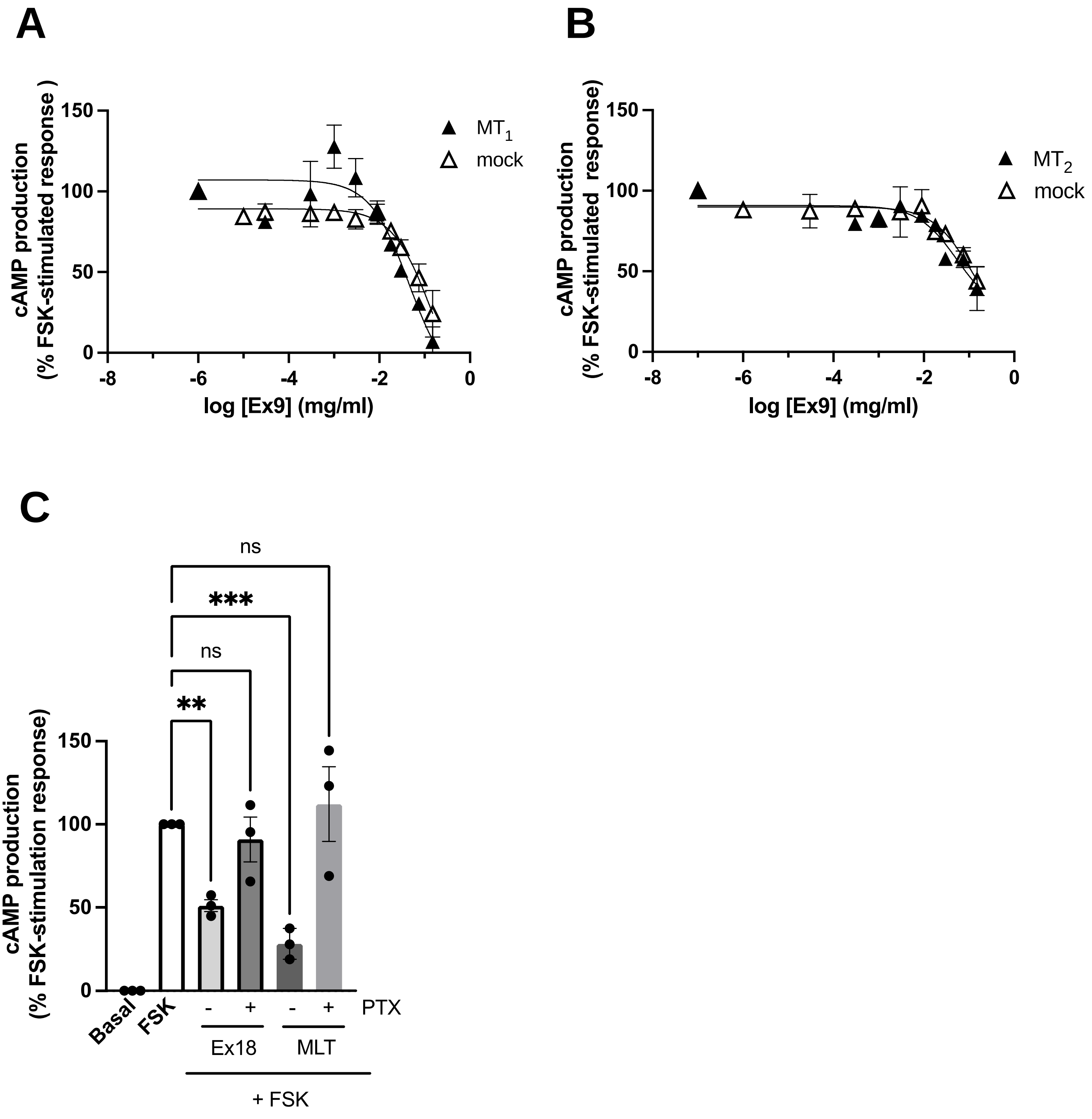

Supp. Figure 3: Effect of selected plant extracts on forskolin-stimulated cAMP production. Effect of increasing concentrations of **Ex9** on HEK293 empty vector transfected cells (mock) *vs.* HEK293 cells expressing MT$_1$ (**A**) or MT$_2$ (**B**) receptors. Data are expressed as mean ± SEM, n = 3. All experiments were performed in triplicates. (**C**) **G_i protein-dependent effect of *Pistacia vera*** in HEK cells expressing MT$_1$ receptors. Cells were pre-treated or not overnight with *Pertussis toxin* (PTX) (10 ng/ml) and stimulated with **MLT** or **Ex18**. Data are normalized to maximal effect of forskolin-stimulated response and are presented as mean ± S.E.M. of 3 experiments performed in triplicates. One way ANOVA (**p < 0.01, ***p < 0.001, ns, not significant).

Supplementary Figure 4

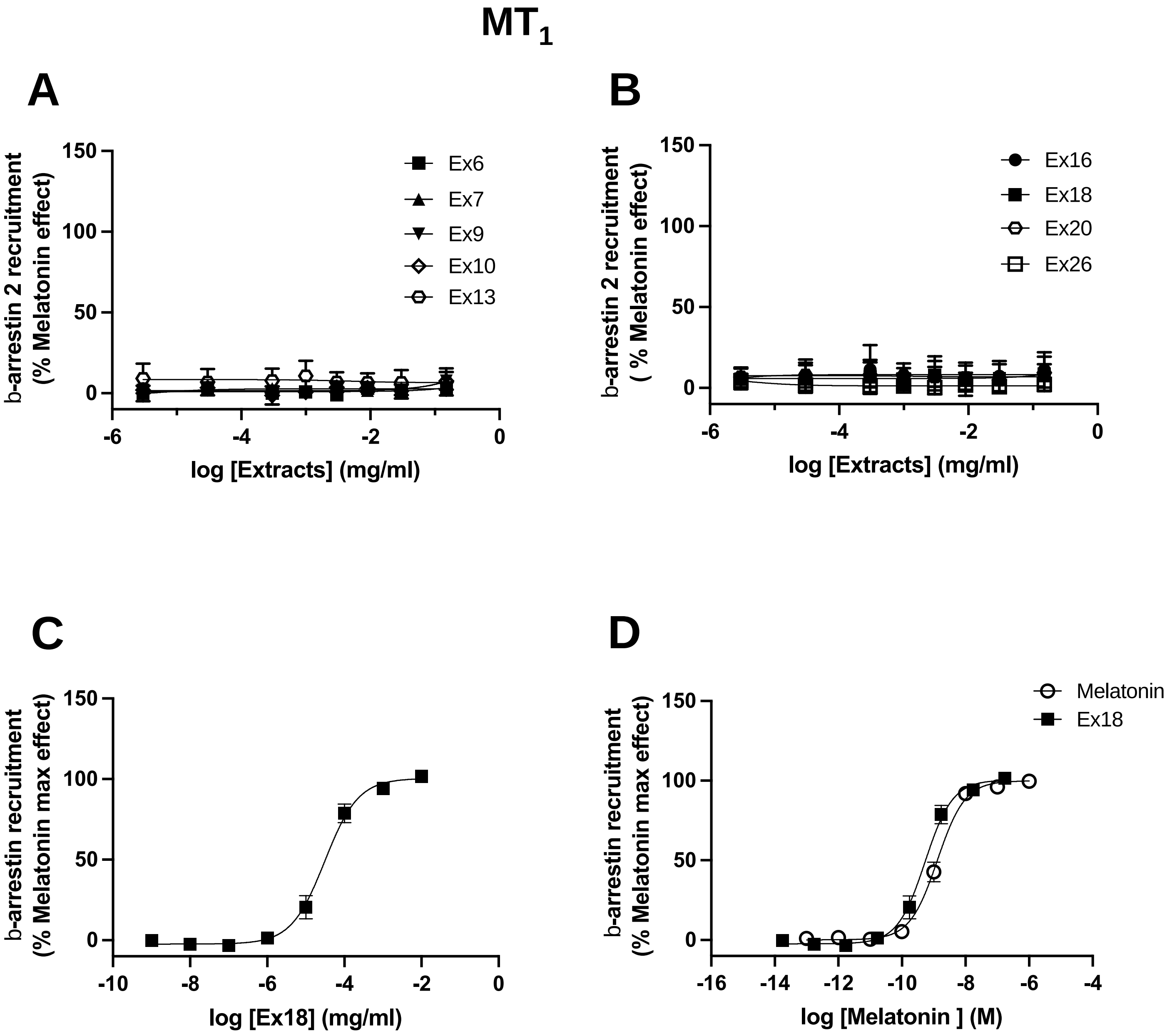

Supp. Figure 4. Effect of selected plant extracts on β-arrestin2 recruitment. Concentration-response curves of selected plant extracts (**A, B**) and **Ex18** (**C, D**) were performed in transient HEK293 cells co-transfected with Rluc-tagged MT_1 receptor and β-arrestin2 tagged with YFP. Data are expressed as percentage of MLT effect and represent 3 experiments performed in triplicates.
